# Introducing national osteopathy practice-based research networks in Australia and New Zealand: an overview to inform future osteopathic research

**DOI:** 10.1038/s41598-020-57918-7

**Published:** 2020-01-21

**Authors:** Amie Steel, Wenbo Peng, David Sibbritt, Jon Adams

**Affiliations:** University of Technology Sydney, Faculty of Health, Australian Research Centre in Complementary and Integrative Medicine, Ultimo, NSW 2006 Australia

**Keywords:** Health services, Public health

## Abstract

Both the Osteopathic Research Innovation Network (ORION) and the Osteopathy Research Connect-New Zealand (ORC-NZ) are practice-based research networks (PBRNs) recently established in Australia and New Zealand. This paper highlights the profile of the osteopaths participating in each PBRN in order to encourage and facilitate further related research in osteopathy. All registered osteopaths in Australia and New Zealand were invited to participate in the ORION and ORC-NZ PBRNs, respectively. This paper presents practice and sociodemographic characteristics of the osteopaths that elected to join the PBRN in their country. A total of 253 registered osteopaths in New Zealand (48.7%) agreed to join ORC-NZ while 992 registered osteopaths in Australia (44.5%) joined ORION. Membership of both PBRNs reflect national geographical spread, and diverse personal and practice characteristics. Combined membership of both PBRNs represents 45.3% of all registered osteopaths in Australasia and 7.7% of the global osteopathic profession. The PBRNs, independently and in combination, hold much potential to advance the evidence-base and capacity of osteopathy research. Both ORION and ORC-NZ PBRNs are powerful, innovative resources available to other interested parties to help conduct further osteopathy research in Australia and New Zealand.

## Introduction

Osteopathy - a manual therapy which follows the principle that structure and function are closely integrated by assessing a person’s musculoskeletal, neurological and visceral systems^1^ - is currently practiced in more than 50 countries worldwide^1^ with a substantial user cohort^2^ especially amongst those seeking care for back pain (8.8%)^3^. In both Australia and New Zealand, osteopathy is an integrated registered health profession^4^, and there is equivalence in the educational requirements to practice osteopathy in accordance with a Trans-Tasman Mutual Recognition Agreement between the professional bodies in both countries^5,6^. Combined, the Australian and New Zealand osteopathic professions represent 17% of the global profession^4^. This is the third highest proportional number of osteopaths, succeeded only by the United States (24.6%) and France (28.9%)^1^.

Despite the widespread practice and use of osteopathy, there remain substantial gaps in the evidence required to best situate osteopathy within contemporary health systems around the world. The quantity and quality of osteopathic research has advanced in recent years and includes pockets of a variety of research interests with particular foci on applied physiology and education^7^. While some research has also investigated specific osteopathic manipulative treatments^8–19^, much of this efficacy research has occurred outside of  an osteopathic clinical setting. For example, a recent review of osteopathic manipulative treatments found only nine whole systems comparative effectiveness studies investigating the outcomes of osteopathic care within a real-world practice environment^20^. Yet, osteopathic treatment is argued to encompass practice and philosophical characteristics that extend beyond osteopathic manipulative treatment alone^21^. There is also range of topics beyond clinical efficacy that relate to the practice and provision of osteopathic care in the community requiring researcher attention, including: a better understanding of the use and users of osteopathy; an exploration of the practice and practices of osteopaths; the positioning of osteopaths alongside other health professionals within different health systems; and the place of osteopathic care within diverse health policy environments^7^. Further infrastructure is needed to build upon the resources and capacity currently available in both Australia and New Zealand to effectively support the breadth and scale of research required to address these and other research topics^22,23^.

A practice-based research network (PBRN) design providing research infrastructure and capacity -building has grown in popularity globally over the last 20 years^24,25^. A PBRN is a group of at least 15 ambulatory practices and/or 15 clinicians that affiliate together and collaborate with academic institutions for conducting research^26,27^. The organisational structure of a PBRN extends beyond any one single study and often encompasses administrative and managerial staff who work alongside the academic contributors and practitioner members to fulfil a shared mission and purpose in research^26^.

**Figure 1:**
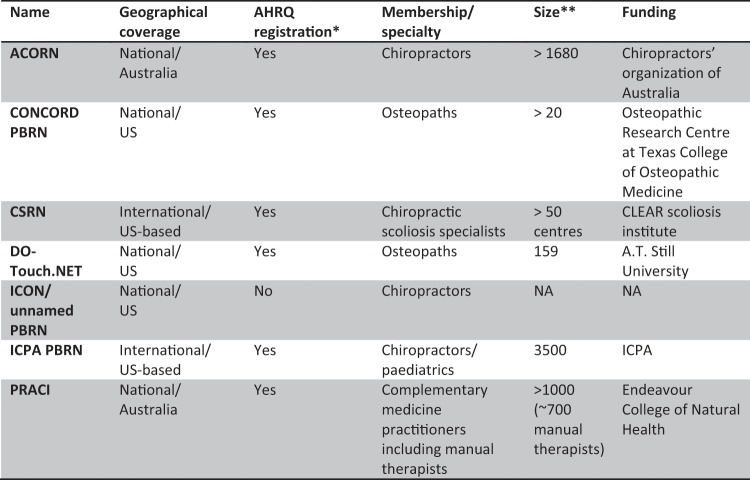
Summary of Manual Therapy Practice-based Research Networks Globally [source: Lee *et al*.^28^]. ACORN, Australian Chiropractic Research Network; AHRQ, Agency for Healthcare Research and Quality; BraveNet, Bravewell Integrative Medicine Research Network; CAM, complementary and alternative medicine; CONCORD, Consortium for Collaborative Osteopathic Research Development; CSRN, CLEAR (Chiropractic, Leadership, Educational, Advancement and Research) Scoliosis Research Network; DO-Touch.NET, Doctors of Osteopathy Treating with Osteopathic Manipulative Medicine: determining its Usefulness in Current Healthcare; ICPA, International Chiropractic Pediatric Association; IM, integrative medicine; NA, not available; PBRN, practice-based research network.

Internationally, a number of PBRNs have been established which encompass clinicians from manual therapy professions (see Fig. 1)^28^. Two of these are national PBRNs of osteopaths in the US; the Consortium for Collaborative Osteopathic Research and Development (CONCORD) coordinated from the Texas College of Osteopathic Medicine (n > 20) and the Doctors of Osteopathy Treating with Osteopathic manipulative medicine (DO-Touch.NET) managed by A.T. Still University (n = 159)^28^. There are also four other PBRNs in Australia that focus upon or include focus upon manual therapists: the Australian Chiropractic Research Network (ACORN) with over 1,680 members^29^; the Practitioner Research and Collaboration Initiative (PRACI) including more than 700 clinicians from a range of manual therapy professions (massage therapy, kinesiology, bowen therapy, myotherapy, and reflexology) alongside other complementary medicine practitioners (over 1,000 members in total)^30^; the Osteopathy Research and Innovation Network (ORION) in Australia and Osteopathy Research Connect-New Zealand (ORC-NZ) in New Zealand. These latter two PBRNs, exclusively focused upon osteopathy and recently established, are overviewed in this paper with a view to helping inform all possible parties of the potential for further collaborations and opportunities to examine a vast range of osteopathy-related issues directly drawing upon these two PBRN initiatives.

## Methodology

### Study objectives

This paper aims to describe the characteristics of the members of two osteopathy practice-based research networks (PBRNs); the ORION PBRN established in Australia and the ORC-NZ PBRN established in New Zealand. While these two PBRNs were established and developed separately, they were designed in part by a common group of senior health researchers and we here provide this description of the projects with a view to helping illustrate how these two PBRNs, both independently but also collectively, constitute significant research infrastructure open to others who wish to answer any of a vast number of osteopathy-related or osteopathy-specific research questions.

### Setting

Recruitment dates differed for each PBRN with ORION recruitment occurring between July 2016 and June 2017 and ORC-NZ recruiting between August and December 2018. Prior to each recruitment period, the research team engaged in each location for up to 12 months to promote to and inform the osteopathic community in each country regarding the forthcoming PBRNs.

### Participants

For both PBRNs, all registered osteopaths in the respective country were invited to participate. Recruitment in Australia was primarily conducted through Osteopathy Australia (OA), the leading professional association for osteopaths. At the time of recruitment there were 1,800 members in OA which represented 80.7% of the 2,230 registered osteopaths in Australia. ORC-NZ recruitment was conducted primarily via the national osteopathy association, Osteopaths New Zealand which had 300 members at the time of recruitment (57.8% of the total 519 registered osteopaths at this time). In addition, the Osteopathic Council of New Zealand, the national registration board for osteopaths, also disseminated invitations to all New Zealand-registered osteopaths regarding participating in the ORC-NZ PBRN.

### Instrument

Data for both PBRNs were collected via an online self-reported questionnaire. The questionnaire was designed with the input from several registered osteopaths in each country – including both professional leaders and those in full-time clinical practice – to ensure face validity. The questionnaire used in each country was matched except for items unique to the local context of each country (e.g. reimbursement models, location of clinical practice). The final instrument included items across three domains: *practitioner characteristics, clinical practice characteristics*, and *clinical management*.

#### Practitioner characteristics

Both groups of respondents in Australia and New Zealand were asked to provide details of their age, gender, highest level of osteopathic qualification, professional association membership, and professional roles (e.g. university teaching, clinical supervision, private practice). ORC-NZ respondents were also invited to identify the country they completed their first osteopathic qualification.

#### Clinical practice characteristics

Both questionnaires collected details about the average number of patient care hours and patient visits per week, practice location/s, co-location and referrals with other health professionals, use of imaging and other diagnostic techniques, and use of electronic records and record-keeping software in clinical practice.

#### Clinical management

Both surveys included items that asked participants about: the public health/health promotion topics (e.g. diet/nutrition, smoking/drugs/alcohol, physical activity/fitness) they discussed with patients as part of their care/management plans; the frequency with which the osteopath treated patients with specific health conditions (e.g. neck pain, low back pain, postural disorders); the frequency with which the osteopath treated different patient subgroups (e.g. children, pregnant women, ethnic populations); and the osteopath’s use of specific techniques/methods (e.g. peripheral joint manipulation, myofascial release, spinal manipulation). All survey items for both projects related to frequency included scaled response items (*never, rarely, sometimes, often*)

After completing the respective survey, participants were invited to provide consent to join the relevant PBRN. This consent included permission for their responses to be identifiably linked to their contact information as part of the respective PBRN database. Those participants who gave such consent provided their first and last name, contact address, email and phone number.

### Statistical analysis

Categorical variables are reported descriptively as frequencies and percentages and continuous variables are reported as a mean with standard deviation. Both cohorts were assessed for representativeness using chi square goodness of fit tests using the data provided through reports from the Osteopathy Board of Australia (for ORION) and Osteopathic Council of New Zealand (for ORC-NZ). All statistical analysis was performed using Stata 14.1 Statistical Analysis software.

### Ethical clearance

This research complies with international standards for ethical conduct as outlined by the Declaration of Helsinki. All participants provided informed consent to participate in the study. Approval for this study was granted by the Human Research Ethics Committee of the University of Technology Sydney.

## Results

A total of 253 osteopaths in New Zealand completed the ORC-NZ survey and agreed to join the ORC-NZ PBRN, representing 48.7% of registered osteopaths in that country at the time of recruitment. The 992 osteopaths in Australia who completed the ORION survey and agreed to join the ORION PBRN represent 44.5% of Australian registered osteopaths at the time of recruitment. Combined, both PBRN practitioner populations represent 45.3% of all registered osteopaths in Australasia.

The ORC-NZ PBRN members were predominantly male (53.0%), had received their first osteopathic qualification in England (51.0%) or New Zealand (39.9%) and reported a Masters (47.2%) or Bachelor (26.2%) degree as their highest osteopathic qualification (see Table 1). Members were an average of 45.4 years old (mean) and reported having been in practice for 15.3 years (mean). Aside from clinical practice, the ORC-NZ PBRN members describe holding a range of other occupational roles including volunteer (18.2%), professional organisation involvement (17.0%) and clinical supervision (of associates [15.8%] and students [7.5%]). Most members described their practice location as ‘urban’ (88.5%).

**Table 1:** Characteristics of members of ORC-NZ (n = 253) and ORION (n = 992).

Practitioner characteristics	ORC-NZ (n = 253)	ORION (n = 992)
	n (%)*	n (%)*
**Gender**		
*Female*	134 (46.6)	576 (58.1)
*Male*	118 (53.0)	416 (41.9)
*Other*	1 (0.4)	0
Age in years [mean (SD)]*	45.4 (12.0)	38.0 (10.9)
**Country qualified**		
*England*	129 (51.0)	—
*New Zealand*	101 (39.9)	—
*Australia*	19 (7.5)	—
*Other*	4 (1.6)	—
*Years in practice* [mean (SD)]*	15.3 (10.6)	11.4 (9.0)
**Highest osteopathic qualification**		
*Diploma*	40 (15.9)	—
*Advanced diploma*	2 (0.8)	9 (0.9)
*Bachelor (or Double Bachelor) degree*	66 (26.2)	214 (21.6)
*Postgraduate certificate or diploma*	17 (6.8)	61 (6.2)
*Masters degree*	119 (47.2)	681 (68.7)
*Other (includes PhD)*	8 (3.2)	27 (2.7)
**Occupational role**		
*University or other teaching*	23 (9.1)	116 (11.7)
*Clinical supervision of students*	19 (7.5)	150 (15.1)
*Clinical supervision of associates*	40 (15.8)	
*Professional organisation involvement*	43 (17.0)	107 (10.8)
*Research*	19 (7.5)	54 (5.4)
*Volunteer*	46 (18.2)	159 (16.0)
**Regionality of primary practice**		
*Urban*	224 (88.5)	820 (82.7)
*Rural*	59 (22.1)	212 (21.4)
*Remote*	4 (1.6)	11 (1.1)

^*^Figures are presented as frequencies and percentages except for continuous variables which are presented as mean and standard deviation.

More ORION PBRN members were female (58.1%) than male (41.9%) with a mean age of 38.0 years old (see Table 1). The substantive majority had been awarded a Master degree as their highest osteopathic qualification (68.7%) while 21.6% had a Bachelor degree. The ORION PBRN members reported being in practice for 11.4 years (mean) although they also undertook other occupational roles; primarily, volunteer (16.0%) and/or clinical supervision (15.1%). Most members reported practicing in an urban location (82.7%).

As seen in Table 2, the majority of ORC-NZ PBRN members indicated their primary practice as located in Auckland (29.6%) while the least number described Northland (4.4%) or Manawatu (4.4%) as their location of practice. ORION members most commonly reported having their primary practice located in Victoria (56.4%), New South Wales (26.5%) or Queensland (9.0%). Northern Territory (0.2%), Australian Capital Territory (1.7%) and South Australian (1.8%) were reported least frequently.

**Table 2:** Location of Primary Practice by Region or State of ORC-NZ (n = 253) and ORION (n = 992).

ORC-NZ (n = 253)		ORION (n = 992)*	
Northland	11 (4.4)	Australian Capital Territory	17 (1.7%)
Auckland	75 (29.6)	New South Wales	263 (26.5%)
Bay of Plenty	27 (10.7)	Northern Territory	2 (0.2%)
Waikato	27 (10.7)	Queensland	89 (9.0%)
Manawatu	11 (4.4)	South Australia	18 (1.8%)
Wellington	30 (11.9)	Tasmania	22 (2.2%)
Nelson	13 (5.1)	Victoria	559 (56.4%)
Canterbury	24 (9.5)	Western Australia	32 (3.2%)
Otago	15 (5.9)		
Other regions	25 (9.9)		

*Participants could select more than one response and as such the sum of the reported frequencies is greater than 100%.

Table 3 describes the clinical practice characteristics of both members of both PBRNs. Members of the ORC-NZ PBRN reported 32.3 patient visits (mean) over 27.5 hours (mean) per week. The majority of ORC-NZ PBRN members practiced in only one clinical location (66.8%) and shared their clinical location with other health professionals (69.8%). Osteopaths were the most common type of health professional co-located with ORC-NZ PBRN members, followed by massage therapists (45.2%) and acupuncturists (38.4%). Most commonly, members of ORC-NZ reported sending referrals to general practitioners (GPs) (90.3%), specialist doctors (76.6%) and acupuncturists (71.4%). Referrals were also received from GPs (92.1%) as well as osteopaths (78.3%) and massage therapists (77.1%). Meanwhile, ORION PBRN members reported consulting with 37.0 (mean) patients over an average of 28.2 (mean) practice hours per week. The majority of ORION PBRN members reported practicing from one clinical location (65.0%), co-located with at least one other osteopath (64.8%), although it was common for a massage therapist (50.5%) to also share the practice site of these osteopaths. GPs (88.5%), podiatrists (65.6%) and massage therapists (67.6%) were sent referrals from ORION PBRN members most frequently, while receiving referrals from GPs (89.3%), massage therapists (76.0%) and/or osteopaths (61.9%) was also reported amongst the ORION PBRN members.

**Table 3:** Clinical practice characteristics of members of ORC-NZ (n = 253) and ORION (n = 992).

Practice characteristics	ORC-NZ (n = 253)	ORION (n = 992)
	Mean (SD)	Mean (SD)
Patient care hours	27.5 (11.1)	28.2 (11.8)
Patient visits	32.3 (22.4)	37.0 (18.3)
	**n (%)**	**n (%)**
Practice in more than one location	84 (33.2)	347 (35.0)
Practice with other health professionals	185 (69.8)	—
*Osteopath*	139 (78.5)	643 (64.8)
*General practitioner*	17 (9.6)	72 (7.3)
*Specialist doctor*	5 (2.8)	31 (3.1)
*Podiatrist*	17 (19.6)	147 (14.8)
*Physiotherapist*	34 (19.2)	144 (14.5)
*Exercise physiologist*	4 (2.3)	124 (12.5)
*Occupational therapist*	4 (2.3)	19 (1.9)
*Psychologist*	34 (19.2)	191 (19.3)
*Massage therapist*	80 (45.2)	501 (50.5)
*Chiropractor*	10 (5.7)	—
*Acupuncturist*	68 (38.4)	188 (19.0)
*Naturopath*	29 (16.4)	193 (19.5)
*Dietician*	6 (3.4)	72 (7.3)
*Nutritionist*	18 (10.2)	78 (7.9)
**Send referrals**		
*Osteopath*	159 (64.1)	506 (51.0)
*General practitioner*	224 (90.3)	878 (88.5)
*Specialist doctor*	190 (76.6)	443 (44.7)
*Podiatrist*	97 (39.1)	651 (65.6)
*Physiotherapist*	113 (45.6)	331 (33.4)
*Exercise physiologist*	20 (8.1)	398 (40.1)
*Occupational therapist*	24 (9.7)	106 (10.7)
*Psychologist*	82 (33.1)	349 (35.2)
*Massage therapist*	160 (64.5)	671 (67.6)
*Chiropractor*	18 (7.3)	—
*Acupuncturist*	177 (71.4)	451 (45.5)
*Naturopath*	105 (42.3)	477 (48.1)
*Dietician*	19 (7.7)	167 (16.8)
*Nutritionist*	49 (19.8)	129 (13.0)
**Receive referrals**		
*Osteopath*	188 (78.3)	614 (61.9)
*General practitioner*	221 (92.1)	886 (89.3)
*Specialist doctor*	79 (32.9)	237 (23.9)
*Podiatrist*	49 (20.4)	471 (47.5)
*Physiotherapist*	114 (47.5)	266 (26.8)
*Exercise physiologist*	15 (6.3)	258 (26.0)
*Occupational therapist*	22 (9.2)	61 (6.1)
*Psychologist*	40 (16.7)	154 (15.5)
*Massage therapist*	185 (77.1)	754 (76.0)
*Chiropractor*	17 (7.1)	—
*Acupuncturist*	129 (53.8)	370 (37.3)
*Naturopath*	96 (40.0)	400 (40.3)
*Dietician*	7 (2.9)	39 (3.9)
*Nutritionist*	19 (7.9)	55 (5.5)

Table 4 reports clinical management practices regarding diagnostic techniques and record-keeping by members of both PBRNs. ORC-NZ PBRN members employed diagnostic imaging ‘sometimes’ (65.2%) or ‘often’ (22.9%) in their clinical care and the most common clinical assessment techniques employed by these NZ osteopaths were orthopaedic testing (96.8%), neurological testing (95.3%), screening questionnaire (88.5%), and cranial neurological testing (68.8%). Approximately more than half of all ORC-NZ PBRN members utilised electronic records for case file management including initial history (54.9%), examination findings (54.6%) and/or subsequent patient visits (56.1%). ORION PBRN members reported ‘sometimes’ (55.9%) or ‘rarely’ (36.2%) using diagnostic imaging in their clinical practice and these Australian-based osteopaths listed orthopaedic testing (97.6%) and neurological testing (92.5%) most frequently for clinical assessment, more so than cranial neurological testing (67.7%) and screening questionnaires (63.8%). Electronic records were reportedly utilised by approximately three quarters of all ORION PBRN members, including for initial history (73.2%), examination findings (74.1%), and/or subsequent patient visits (76.5%).

**Table 4:** Clinical management practices regarding diagnostic techniques and record-keeping of members of ORC-NZ (n = 253) and ORION (n = 992).

	ORC-NZ (n = 253)	ORION (n = 992)
**Diagnostic imaging**		
*Never*	1 (0.4)	6 (0.6)
*Rarely*	29 (11.5)	359 (36.2)
*Sometimes*	165 (65.2)	554 (55.9)
*Often*	58 (22.9)	73 (7.4)
**Clinical assessment techniques**		
*Orthopaedic testing*	245 (96.8)	968 (97.6)
*Clinical assessment algorithm*	77 (30.4)	468 (47.2)
*Neurological testing*	241 (95.3)	918 (92.5)
*Screening questionnaire*	224 (88.5)	633 (63.8)
*Cranial neurological testing*	174 (68.8)	672 (67.7)
**Electronic records**		
*Initial history*	139 (54.9)	726 (73.2)
*Examination findings*	138 (54.6)	735 (74.1)
*Subsequent patient visits*	142 (56.1)	759 (76.5)
*Never*	110 (43.5)	232 (23.4)

The conditions and populations treated by members of both osteopathy PBRNs are presented in Table 5. Neck pain (98.4%), low back pain (98.4%), thoracic spine and rib pain (88.9%), headache disorders (86.6%) and shoulder musculoskeletal disorders were reported by the most ORC-NZ PBRN members as being treated ‘often. While 47.3% of ORC-NZ PBRN members indicated they ‘rarely’ or ‘never’ treated non-musculoskeletal disorders, 36.5% ‘sometimes’ and 16.3% ‘often’ treated this category of condition. The most common populations treated by ORC-NZ PBRN members were older people (65 years and over) (66.4%), people with work-related injuries (64.8%) and people with sports-related injuries (51.8%). Patients claiming Accident Compensation Corporation (ACC) reimbursement were also treated by the majority of ORC-NZ PBRN members (88.1%). The majority of ORC-NZ PBRN members ‘sometimes’ treated patients identifying as Maori (55.7%), pregnant women (53.0%), and children between the ages of 4 and 18 years old (51.2%). Younger children (up to 3 years old) were ‘rarely’ or ‘never’ treated by 40.8% of ORC-NZ PBRN members.

**Table 5:** Conditions and populations treated by members of ORC-NZ (n = 253) and ORION (n = 992).

	ORC-NZ (n = 253)				ORION (n = 992)			
	*Often*	*Sometimes*	*Rarely*	*Never*	*Often*	*Sometimes*	*Rarely*	*Never*
	n (%)	n (%)	n (%)	n (%)	n (%)	n (%)	n (%)	n (%)
**Conditions treated**								
*Neck pain*	249 (98.4)	4 (1.6)	0 (0.0)	0 (0.0)	971 (98.0)	20 (2.0)	0 (0.0)	0 (0.0)
*Thoracic spine and rib pain*	225 (88.9)	27 (10.7)	1 (0.4)	0 (0.0)	909 (91.7)	80 (8.1)	2 (0.2)	0 (0.0)
*Low back pain*	249 (98.4)	4 (1.6)	0 (0.0)	0 (0.0)	977 (98.7)	10 (1.0)	1 (0.1)	2 (0.2)
*Hip pain*	173 (68.4)	76 (30.0)	3 (1.2)	1 (0.4)	744 (75.2)	236 (23.8)	8 (0.8)	2 (0.2)
*Knee musculoskeletal disorders*	126 (49.8)	116 (45.9)	11 (4.4)	0 (0.0)	491 (49.7)	456 (46.2)	38 (3.9)	3 (0.3)
*Ankle musculoskeletal disorders*	97 (38.3)	120 (47.4)	36 (14.0)	0 (0.0)	333 (33.7)	501 (50.7)	150 (15.2)	5 (0.5)
*Foot musculoskeletal disorders*	74 (29.3)	119 (47.0)	60 (23.7)	0 (0.0)	294 (29.7)	484 (48.9)	207 (20.9)	5 (0.5)
*Shoulder musculoskeletal disorders*	207 (81.8)	45 (17.8)	1 (0.4)	0 (0.0)	801 (81.0)	176 (17.8)	10 (1.0)	2 (0.2)
*Elbow musculoskeletal disorders*	60 (23.8)	140 (55.6)	49 (19.4)	3 (1.2)	251 (25.5)	558 (56.6)	170 (17.2)	7 (0.7)
*Wrist musculoskeletal disorders*	45 (17.9)	126 (50.2)	79 (31.5)	1 (0.4)	188 (19.0)	469 (47.4)	325 (32.9)	7 (0.7)
*Hand musculoskeletal disorders*	33 (13.2)	100 (39.8)	114 (45.4)	4 (1.6)	121 (12.3)	352 (35.7)	482 (48.9)	30 (3.1)
*Postural disorders*	132 (52.6)	89 (35.5)	30 (12.0)	0 (0.0)	675 (68.3)	261 (26.4)	52 (5.3)	1 (0.1)
*Degenerative spine disorders*	123 (49.0)	95 (37.9)	31 (12.4)	2 (0.8)	599 (60.6)	324 (32.8)	66 (6.7)	0 (0.0)
*Headache disorders*	219 (86.6)	33 (13.0)	1 (0.4)	0 (0.0)	892 (90.1)	95 (9.6)	2 (0.2)	1 (0.1)
*Migraine disorders*	104 (41.1)	126 (49.8)	22 (8.7)	1 (0.4)	400 (40.5)	518 (52.4)	69 (7.0)	1 (0.1)
*Spinal health maintenance*	121 (48.0)	98 (38.9)	31 (12.3)	2 (0.8)	458 (46.4)	378 (38.3)	136 (13.8)	16 (1.6)
*Chronic or persistent pain*	127 (50.2)	108 (42.7)	18 (7.1)	0 (0.0)	630 (63.7)	310 (31.3)	47 (4.8)	2 (0.2)
*Tendinopathies*	76 (30.2)	129 (51.2)	44 (17.5)	3 (1.2)	410 (41.5)	477 (48.2)	96 (9.7)	6 (0.6)
*Temporomandibular joint (TMJ) disorders*	35 (13.8)	116 (45.9)	98 (38.7)	4 (1.6)	183 (18.5)	504 (51.0)	291 (29.5)	10 (1.0)
*Non-musculoskeletal disorders*	41 (16.3)	92 (36.5)	77 (30.6)	42 (16.7)	126 (12.9)	262 (26.7)	318 (32.5)	274 (28.0)
**Populations treated**								
*Children (up to 3 years)*	72 (28.8)	76 (30.4)	51 (20.4)	51 (20.4)	156 (15.8)	217 (22.0)	304 (30.8)	311 (31.5)
*Children (4 to 18 years)*	89 (35.3)	129 (51.2)	33 (13.1)	1 (0.4)	270 (27.3)	545 (55.0)	168 (17.0)	8 (0.8)
*Older people (65 year or over)*	168 (66.4)	81 (32.0)	4 (1.6)	0 (0.0)	572 (57.7)	369 (37.2)	48 (4.8)	2 (0.2)
Maori/Australian Indigenous*	39 (15.4)	141 (55.7)	71 (28.0)	2 (0.8)	7 (0.7)	105 (10.6)	547 (55.3)	331 (33.4)
*Pregnant women*	75 (29.6)	134 (53.0)	43 (17.0)	1 (0.4)	344 (34.7)	534 (53.9)	108 (10.9)	5 (0.5)
*People with sports-related injuries*	131 (51.8)	104 (41.1)	18 (7.1)	0 (0.0)	501 (50.6)	432 (43.6)	53 (5.4)	4 (0.4)
*People with work-related injuries*	164 (64.8)	81 (32.0)	8 (3.2)	0 (0.0)	—	—	—	—
*People with traffic-related injuries*	68 (26.9)	136 (53.8)	45 (17.8)	4 (1.6)	—	—	—	—
*People receiving post-surgical rehabilitation*	35 (13.8)	125 (49.4)	84 (34.3)	6 (2.4)	79 (8.0)	456 (46.1)	396 (40.0)	58 (5.9)
*Non-English speaking ethnic groups*	12 (4.8)	60 (23.8)	105 (41.7)	75 (29.8)	33 (3.3)	153 (15.5)	457 (46.2)	346 (35.0)
*Treat patients with ACC reimbursement*	223 (88.1)	23 (9.1)	0 (0.0)	7 (2.8)	—	—	—	—

*Data reports treatment of Maori populations for ORC-NZ and Australian indigenous populations for ORION.

ORION PBRN members often treated low back pain (98.7%), neck pain (98.0%), thoracic spine and rib pain (91.7%), headache disorders (90.1%) and shoulder musculoskeletal disorders (81.0%). Non-musculoskeletal disorders were ‘never’ or ‘rarely’ treated by 60.5% of ORION PBRN members. More than half of the ORION membership reported ‘often’ treating older people (65 years and over) (57.7%) and people with sports-related injuries (50.6%), and ‘sometimes’ treating children between the ages of 4 and 18 years old (55.0%) and pregnant women (53.9%). Australian indigenous people were ‘rarely’ (55.3%) or ‘never’ (33.4%) treated by ORION PBRN members, as were patients from non-English speaking ethnic groups (rarely: 46.2%; never: 35.0%) or children younger than three years old (rarely: 30.8%; never: 31.5%).

The most common topics discussed with patients as part of their care plan by ORC-NZ PBRN members were physical activity/fitness (86.9%), stress management (53.8%) and occupational health and safety (43.3%) (see Table 6). Other topics such as smoking, drugs and alcohol (52.8%), medications (51.8%), nutritional supplements (47.4%), and diet/nutrition (46.8%) were ‘sometimes’ discussed by substantial numbers of ORC-NZ PBRN members. A range of techniques were reported by ORC-NZ PBRN members as employed in their treatment of patients, with the most common being soft issue techniques (87.4%), exercise prescription or advice (78.3%), high velocity-low amplitude/spinal manipulation (61.3%), myofascial release (60.9%), muscle energy techniques (59.9%), peripheral joint manipulation (53.8%) and cranial techniques (50.6%). ORION PBRN members reported ‘often’ discussing physical activity/fitness (89.4%) and occupational health and safety (51.2%) with their patients. Other topics such as medications (48.0%), diet/nutrition (47.0%), smoking/drugs/alcohol (45.9%) and nutritional supplements (45.0%) were also ‘sometimes’ discussed with patients by ORION PBRN members. The most common techniques used by ORION PBRN members when treating patients were soft tissue techniques (85.7%), muscle energy techniques (79.5%), exercise prescription or advice (74.0%), visceral techniques (70.0%), high velocity-low amplitude/spinal manipulation (63.8%) and myofascial release (61.8%).

**Table 6:** Features of the clinical treatments and care plans of members of ORC-NZ (n = 253) and ORION (n = 992).

	ORC-NZ (n = 253)				ORION (n = 992)			
	*Often*	*Sometimes*	*Rarely*	*Never*	*Often*	*Sometimes*	*Rarely*	*Never*
**Topics discussed with patients**								
*Diet/Nutrition*	104 (41.3)	118 (46.8)	29 (11.5)	1 (0.4)	375 (37.9)	465 (47.0)	142 (14.3)	8 (0.8)
*Smoking/Drugs/Alcohol*	32 (12.8)	132 (52.8)	80 (32.0)	6 (2.4)	179 (18.1)	454 (45.9)	324 (32.7)	33 (3.3)
*Physical activity/fitness*	219 (86.9)	29 (11.5)	4 (1.6)	0 (0.0)	886 (89.4)	99 (10.0)	6 (0.6)	0 (0.0)
*Occupational health and safety*	109 (43.3)	101 (40.1)	36 (14.3)	6 (2.4)	506 (51.2)	374 (37.8)	95 (9.6)	14 (1.4)
*Pain counselling*	55 (22.0)	106 (42.4)	79 (31.6)	10 (4.0)	264 (26.6)	411 (41.5)	266 (26.8)	50 (5.1)
*Stress management*	141 (53.8)	103 (39.3)	18 (6.9)	0 (0.0)	489 (49.4)	410 (41.5)	85 (8.6)	5 (0.5)
*Nutritional supplements*	66 (26.3)	119 (47.4)	52 (20.7)	14 (5.6)	252 (25.4)	446 (45.0)	247 (24.9)	46 (4.6)
*Medications (including for pain/inflammation)*	92 (36.4)	131 (51.8)	22 (8.7)	8 (3.2)	391 (39.5)	475 (48.0)	115 (11.6)	9 (0.9)
**Treatment techniques used**								
*Strain/Counterstrain*	66 (26.9)	76 (31.0)	61 (24.9)	42 (17.1)	420 (42.4)	324 (32.7)	180 (18.2)	66 (6.7)
*Muscle energy techniques*	151 (59.9)	68 (26.9)	24 (9.5)	9 (3.6)	788 (79.5)	154 (15.5)	34 (3.4)	15 (1.5)
*High velocity low amplitude/spinal manipulation*	155 (61.3)	45 (17.8)	30 (11.9)	23 (9.1)	632 (63.8)	231 (23.3)	86 (8.7)	42 (4.2)
*Peripheral joint manipulation*	136 (53.8)	72 (28.5)	31 (12.3)	14 (5.5)	393 (39.7)	347 (35.1)	212 (21.4)	37 (3.7)
*Soft tissue techniques*	221 (87.4)	15 (5.9)	11 (4.4)	6 (2.4)	848 (85.7)	85 (8.6)	46 (4.7)	11 (1.1)
*Myofascial release*	154 (60.9)	62 (24.5)	23 (9.1)	14 (5.5)	612 (61.8)	266 (26.9)	79 (8.0)	33 (3.3)
*Cranial techniques*	128 (50.6)	68 (26.9)	31 (12.3)	26 (10.3)	233 (23.5)	219 (22.1)	213 (21.5)	325 (32.8)
*Facilitated positional release*	62 (24.8)	78 (31.2)	49 (19.6)	61 (24.4)	166 (16.8)	298 (30.1)	314 (31.8)	211 (21.3)
*Needling techniques*	12 (4.7)	4 (1.6)	4 (1.6)	233 (92.1)	234 (23.6)	165 (16.7)	51 (5.2)	540 (54.6)
*Visceral techniques*	58 (22.9)	98 (38.7)	69 (27.3)	28 (11.1)	98 (70.0)	272 (27.5)	411 (41.5)	210 (21.2)
*Lymphatic pump*	24 (9.5)	93 (36.8)	97 (38.3)	39 (15.4)	84 (8.5)	316 (31.9)	415 (41.9)	176 (17.8)
*Autonomic balancing*	52 (20.8)	59 (23.6)	59 (23.6)	80 (32.0)	157 (15.9)	190 (19.2)	216 (21.8)	427 (43.1)
*Biodynamic techniques*	49 (19.4)	34 (13.5)	47 (18.7)	122 (48.4)	155 (15.6)	94 (9.5)	156 (15.7)	586 (59.1)
*Functional techniques*	115 (45.5)	85 (33.6)	42 (16.6)	11 (4.4)	270 (27.3)	335 (33.8)	251 (25.3)	135 (13.6)
*Balanced ligamentous tension/Ligamentous articular strain*	120 (47.8)	67 (26.7)	46 (18.3)	18 (7.2)	349 (35.2)	279 (28.2)	213 (21.5)	150 (15.1)
*Exercise prescription or advice*	198 (78.3)	46 (18.2)	8 (3.2)	1 (0.4)	733 (74.0)	218 (22.0)	35 (3.5)	4 (0.4)
*Chapmans reflexes*	10 (4.0)	18 (7.2)	54 (21.5)	169 (67.3)	24 (2.4)	78 (7.9)	190 (19.2)	698 (70.5)
*Shockwave therapy*	2 (0.8)	6 (2.4)	2 (0.8)	242 (96.0)	18 (1.8)	35 (3.5)	27 (2.7)	910 (91.9)
*Ultrasound therapy*	1 (0.4)	3 (1.2)	3 (1.2)	245 (97.2)	27 (2.7)	32 (3.2)	50 (5.1)	880 (89.0)
*TENS or other electrotherapy*	6 (2.4)	3 (1.2)	6 (2.4)	234 (94.0)	19 (1.9)	25 (2.5)	67 (6.8)	879 (88.8)
*Instrument-assisted manipulative techniques*	0 (0.0)	2 (0.8)	3 (1.2)	247 (98.0)	2 (0.2)	11 (1.1)	20 (2.0)	956 (96.7)
*Instrument-assisted soft tissue mobilisation*	2 (0.8)	7 (2.8)	9 (3.6)	234 (92.9)	12 (1.2)	29 (2.9)	37 (3.7)	912 (92.1)
*Trigger point therapy*	73 (29.1)	75 (30.0)	43 (17.1)	60 (23.9)	258 (26.1)	353 (35.7)	184 (18.6)	195 (19.7)
*Sports taping*	25 (9.9)	57 (22.6)	85 (33.7)	85 (33.7)	122 (12.3)	330 (33.3)	311 (31.4)	227 (22.9)
*Breathing retraining*	63 (25.2)	108 (43.2)	54 (21.6)	25 (10.0)	—	—	—	—

## Discussion

This paper presents an overview of the practitioner and practice characteristics of those osteopaths who are foundational members of either the ORION PBRN or ORC-NZ PBRN with a view to encouraging and facilitating further research drawing upon each or both of the PBRN baseline membership databases. These two PBRN initiatives represent the two most extensive voluntary PBRNs in the world with regards to coverage of a health profession. Such recruitment success, exceeding the proportion of the chiropractic profession recruited to the ACORN PBRN in Australia (36%)^29^, is built upon memberships of 48.7% and 45.3% of the total profession in New Zealand and Australia respectively. By establishing these osteopathy PBRNs (sharing a similar baseline instrument design contextualised to each local setting), the infrastructure of each PBRN capitalises on the Trans-Tasman Mutual Recognition in place between both countries^5,6^ and provides the opportunity for Australasian osteopathy research projects maximising the value of each individual PBRN. As the cumulative membership of the two PBRNs represents 7.7% of the global osteopathic profession^1^, these large scale program initiatives hold much potential to facilitate future osteopathy research.

### Future directions

Both ORC-NZ and ORION have been established in line with the common characteristics of PBRNs^26^. As such, each PBRN (both independently and when considered in combination) holds much potential to significantly advance the evidence-base and research capacity of and within osteopathy on a global scale. Many PBRNs (across many professions/areas of health care practice and geographical locations) draw upon ‘big data’ study designs (*registry model*) - whereby initial data collection is focused on establishing a centralized, coordinated patient record management system^31^ - limiting the ability to develop further projects sensitive to different clinical settings/circumstances or the evolving needs of grass-roots practitioners and the wider profession^32–34^. In contrast, for both the ORION and ORC-NZ PBRN a *sub-study model* was employed – a design successfully founded and employed in other Australian PBRNs^29,30^. This approach to PBRN design – whereby initial data collection is focused exclusively on practitioner-relevant information collected via self-report aimed at establishing a practitioner PBRN database - facilitates the conduct of further nested studies addressing a variety of research questions and potentially utilising diverse research designs. Both ORION and ORC-NZ PBRNs are actively open for sub-study submissions by any appropriately qualified research teams. Sub-study guidelines and application forms can be located via the official ORION and ORC-NZ PBRN websites (please see www.orion-arccim.com and www.orcnz-arccim.org for further details). In order to help encourage use of the two osteopathy PBRNs for sub-study recruitment, below we outline some examples (not exhaustive) of the types of research and research designs that may be employed through further empirical investigation nested within and/or building upon the ORC-NZ and ORION PBRN programs.

#### Pragmatic clinical research

For clinical trials to be of greatest value to the osteopathic profession requires they be both rigorous and practice-relevant/informed^35^. Unfortunately, there are a number of challenges in conducting pragmatic trials not the least being access to both clinics and clinicians^36^. The ORION and ORC-NZ PBRNs have the potential to facilitate impressive researcher access to both osteopathy clinics and individual osteopaths who may be interested in assisting with a pragmatic clinical trial study. As such, each of the two osteopathy PBRNs offer the osteopathic research community a welcome opportunity to advance beyond single-setting study designs which have to date tended to dominate clinical research in osteopathy^37^. Multi-centre clinical trials are well-regarded as they provide additional rigour to the study design, produce increased sensitivity of data regarding effect size, and may compensate for issues regarding clinician blinding^38^. It is also recommended that clinicians delay integrating new practices into their clinical decision-making until there is evidence drawn from multi-centre clinical trials to support the practice^38,39^. The shared characteristics of ORION and ORC-NZ add further strength to any potential clinical trial research using these PBRNs as they can support pragmatic multi-centre trials conducted in more than one country, thereby adding to the external validity of any findings.

#### Observational research

The ORION and ORC-NZ PBRNs can accommodate retrospective, prospective or cross-sectional observational sub-studies targeting osteopaths or their patients. Due to the substantive number of members in the two PBRNs, the total volume of patients accessible via the networks is considerable and presents the potential for statistically powerful sub-studies, with large samples of participants, to be recruited over a relatively short timeframe. Furthermore, sub-study researchers looking to recruit via one or both of the osteopathy PBRNs are able to employ study designs which rely upon successful recruitment of only a small number of patients per site across a large number of PBRN member sites, thereby not only reaching a statistically powerful sample size with ease but also maximising the generalisability of the data^40^. Researchers may also choose to use ORC-NZ or ORION to sample osteopaths as participants to explore clinical observations, attitudes and beliefs. Findings from such studies can be used to: inform the development of clinical trials; identify knowledge or skill gaps that may benefit from knowledge translation interventions; and mobilise knowledge developed through clinical experience which may assist with the understanding and management of conditions commonly seen within osteopathic practice^41,42^.

#### Qualitative research

The sub-study design of both ORC-NZ and ORION affords the flexibility to accommodate research projects employing qualitative research methods. One of the distinguishing features of qualitative research is the preference for investigating naturally occurring behaviour and settings or at least the direct accounts of participants involved in such naturally occurring settings^43^. This feature of qualitative research is particularly well supported by the two osteopathy PBRNs given they are characterised by a focus upon grass-roots providers delivering care to the community from clinical practices. Researchers can use ORION and ORC-NZ to explore, through qualitative research fieldwork, a deeper understanding of the meanings and experiences of osteopaths and their patients (plus other related stakeholders as necessary) with regards to a wide range of pertinent topics^44^.

#### Accumulation of case reports and single-subject research

Case reports can be described as a form of descriptive research that seeks to identify explanatory patterns for phenomena^45^. Data captured from individual clinical cases through case reports can form the basis of new directions in clinical research^46^. Case reports can be undertaken as prospective case series through which a researcher can work with osteopaths with a special interest in the management of a particular condition or in the application of a specific osteopathic technique. Data for a case series can be collected retrospectively by osteopaths using data extraction tables provided by a research team to help summarise information of existing case records^46^. In contrast to case reports, single subject research, including N of 1 trials, are characterised by repeated measures of an observable and clinically-relevant target behaviour throughout at least one pre-treatment (baseline) and intervention phase^45^. While the PBRN infrastructure of ORION and ORC-NZ is not a requirement for either case reports or single-subject research, these networks do nevertheless offer researchers the ability to collect case series and multi-person N of 1 studies more easily and with less burden on individual clinicians. This is particularly the case for rarely treated conditions (e.g. hand disorders), specific populations (e.g. non-English speaking ethnic groups) or specialised treatments (e.g Chapmans reflexes, needling techniques or visceral techniques) whereby researchers may be able to more effectively identify relevant cases for inclusion in their study. Furthermore, inviting osteopaths from multiple sites to share case reports and assist with data collection for single-subject research can strengthen the rigour of the data and robustness of the findings^46,47^.

## Conclusions

Both ORION and ORC-NZ PBRNs are powerful and innovative resources that will help advance osteopathy research in Australia and New Zealand as well as help facilitate collaborations with interested practitioners and researchers further afield. When combined, these two osteopathy PBRNs represent the largest coverage of any health profession within any existing voluntary PBRN and as such signify a substantial opportunity for osteopathy and osteopathy researchers. Nevertheless, it remains that these innovative resources require extensive engagement from practitioners, professional associations, methodologists and others in order to realise their full potential and there are always challenges in engaging large numbers of clinicians in the research process over time. However, given the initial support and investment of both Osteopathy Australia and Osteopaths New Zealand and the impressive response of so many osteopaths across both Australia and New Zealand to the initial calls for PBRN participation, there is reason to remain optimistic that both ORION and ORC-NZ can help grow osteopathy research and help advance a research culture that is rigorous, clinically relevant and reflects the diversity and nature of osteopathic practice across Australasia.
